# Supplementary material for: Comparison of blood and urine concentrations of equol by LC‒MS/MS method and factors associated with equol production in 466 Japanese men and women
Source: PLoS One. 2024 Mar 27;19(3):e0288946. doi: 10.1371/journal.pone.0288946 (PMC10971664; doi:10.1371/journal.pone.0288946)
Supplement: S1 File — (PDF) [file pone.0288946.s001.pdf]

Consent on participating in the research on advanced medical care and life style related diseases

Explanation
Upon your consent, we will conduct the following.

Medical check-up

・Medical check-up will be performed on the year of agreement and following years.

・Questionnaire, blood collection and imaging tests will be performed in addition to basic medical check-up.

・The remaining specimens (blood, urine, tissues, etc.) from the medical check-up will be stored and used for future research.

・All the medical check-up information will be used for this research purpose.

・Stored data and specimens will also be used for this research purpose.

・The examination items may subject to change in accordance with the circumstances of the research.

・The participants need to provide blood and tissue specimens for genomic research and storage for future research.

・Research data can be used for secondary purposes.

・The research items will not be reported to individuals.

・The participants shall not own the intellectual property produced by this research.

・Midtown Clinic is generally responsible for compensation of any adverse events due to participating in this research.

Protection of personal information (confidentiality)

・Your personal information will be strictly protected by the Kyoto University Hospital.

・Your DNA will be stored at the biobank in accordance with its laws and regulations.

Regarding the storage, analysis, attribution and announcement of results obtained from blood, urine, medical check-up data and follow-up survey data

・the Kyoto University Hospital will store the questionnaire items, test results, blood, uring, tissue, DNA within the research period, and will continue to do so as long as the research is going on.

・When the research is completed, there will be prior consultation within the Kyoto University Hospital with regards to storage of the above materials.

・The results of the research and survey items will be used in research related to the Kyoto University, as well as will be announced to the outside in the future, in compliance with the protection of personal information.

・The research results will be written as articles, announced to the public in compliance with personal data protection.

Participation is on your own free will and there is no disadvantage if you refuse to participate.
You can withdraw the consent any time.

TO: Medical Director, Kyoto University Hospital

Upon comprehending all the above-mentioned facts, I gave consent on participating in the research on advanced medical care and life style related diseases.

Name: ______________________________ In Furigana: __________________________

Date of signature: ____________________

Explained by _________________________ Date: ________________________
