# Supplementary figures and images for: Comparison of blood and urine concentrations of equol by LC‒MS/MS method and factors associated with equol production in 466 Japanese men and women
Source: PLoS One. 2024 Mar 27;19(3):e0288946. doi: 10.1371/journal.pone.0288946 (PMC10971664; doi:10.1371/journal.pone.0288946)

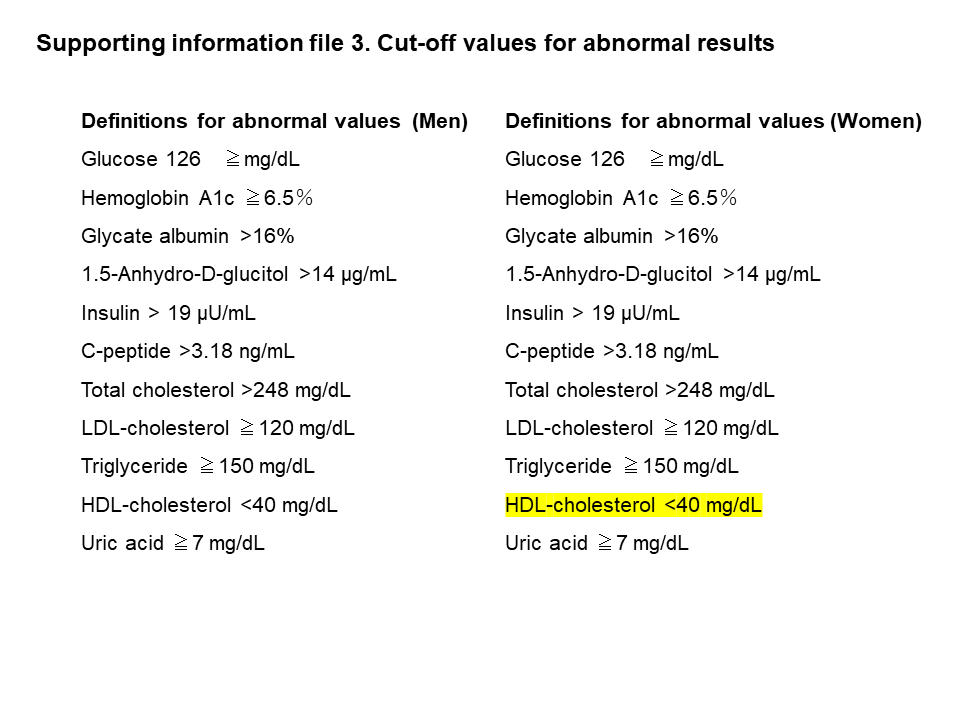

Supplement: S3 File — (TIF) [file pone.0288946.s003.tif]
