# Supplementary material for: Comparison of blood and urine concentrations of equol by LC‒MS/MS method and factors associated with equol production in 466 Japanese men and women
Source: PLoS One. 2024 Mar 27;19(3):e0288946. doi: 10.1371/journal.pone.0288946 (PMC10971664; doi:10.1371/journal.pone.0288946)
Supplement: Attachment — Submitted filename: Response to reviewers_PONE-D-23-20874.docx [file pone.0288946.s005.docx]

Journal Requirements:

When submitting your revision, we need you to address these additional requirements.

1. Please ensure that your manuscript meets PLOS ONE's style requirements, including those for file naming. The PLOS ONE style templates can be found at

https://journals.plos.org/plosone/s/file?id=wjVg/PLOSOne_formatting_sample_main_body.pdf and

https://journals.plos.org/plosone/s/file?id=ba62/PLOSOne_formatting_sample_title_authors_affiliations.pdf

*Thank you for your references. We have revised the manuscript to meet the requirements.*

2. Note from Emily Chenette, Editor in Chief of PLOS ONE, and Iain Hrynaszkiewicz, Director of Open Research Solutions at PLOS: Did you know that depositing data in a repository is associated with up to a 25% citation advantage (https://doi.org/10.1371/journal.pone.0230416)? If you’ve not already done so, consider depositing your raw data in a repository to ensure your work is read, appreciated and cited by the largest possible audience. You’ll also earn an Accessible Data icon on your published paper if you deposit your data in any participating repository (https://plos.org/open-science/open-data/#accessible-data).

*Thank you for your advice. We will deposit the raw data there when the manuscript was accepted.*

3. Please provide additional details regarding participant consent. In the ethics statement in the Methods and online submission information, please ensure that you have specified (1) whether consent was informed and (2) what type you obtained (for instance, written or verbal, and if verbal, how it was documented and witnessed). If your study included minors, state whether you obtained consent from parents or guardians. If the need for consent was waived by the ethics committee, please include this information.

*Thank you for your advice. We have added those points in the ethical consideration section.*

If you are reporting a retrospective study of medical records or archived samples, please ensure that you have discussed whether all data were fully anonymized before you accessed them and/or whether the IRB or ethics committee waived the requirement for informed consent. If patients provided informed written consent to have data from their medical records used in research, please include this information

*Thank you for your advice. We have added those points in the ethical consideration section.*

Once you have amended this/these statement(s) in the Methods section of the manuscript, please add the same text to the “Ethics Statement” field of the submission form (via “Edit Submission”).

*Thank you for your advice. We have added those points in the submission form.*

For additional information about PLOS ONE ethical requirements for human subjects research, please refer to http://journals.plos.org/plosone/s/submission-guidelines#loc-human-subjects-research.

4. Thank you for stating the following financial disclosure:

"NO. The funders had no role in study design, data collection and analysis, decision to publish, or preparation of the manuscript."

At this time, please address the following queries:

a)	Please clarify the sources of funding (financial or material support) for your study. List the grants or organizations that supported your study, including funding received from your institution.

b)	State what role the funders took in the study. If the funders had no role in your study, please state: “The funders had no role in study design, data collection and analysis, decision to publish, or preparation of the manuscript.”

c)	If any authors received a salary from any of your funders, please state which authors and which funders.

d)	If you did not receive any funding for this study, please state: “The authors received no specific funding for this work.”

Please include your amended statements within your cover letter; we will change the online submission form on your behalf.

*Thank you for your advice. We have added those points in the cover letter.*

5. In your Data Availability statement, you have not specified where the minimal data set underlying the results described in your manuscript can be found. PLOS defines a study's minimal data set as the underlying data used to reach the conclusions drawn in the manuscript and any additional data required to replicate the reported study findings in their entirety. All PLOS journals require that the minimal data set be made fully available. For more information about our data policy, please see http://journals.plos.org/plosone/s/data-availability.

*Thank you for your advice. We will deposit the raw data there when the manuscript was accepted.*

Upon re-submitting your revised manuscript, please upload your study’s minimal underlying data set as either Supporting Information files or to a stable, public repository and include the relevant URLs, DOIs, or accession numbers within your revised cover letter. For a list of acceptable repositories, please see http://journals.plos.org/plosone/s/data-availability#loc-recommended-repositories. Any potentially identifying patient information must be fully anonymized.

*Thank you for your advice. We have added the data set as Supporting information.*

Important: If there are ethical or legal restrictions to sharing your data publicly, please explain these restrictions in detail. Please see our guidelines for more information on what we consider unacceptable restrictions to publicly sharing data: http://journals.plos.org/plosone/s/data-availability#loc-unacceptable-data-access-restrictions. Note that it is not acceptable for the authors to be the sole named individuals responsible for ensuring data access.

We will update your Data Availability statement to reflect the information you provide in your cover letter.

Reviewer's Responses to Questions

**Comments to the Author**

Reviewer #1: This cross-sectional study calculated a cutoff value for the benefit of equol-producers in Japanese men and women. While it is worth reporting that the cutoff was the same for both sexes (-1.42), the authors need to consider the following points.

Major points
1. The authors emphasize that there was a favorable trend in the equol producers, but this is not evident from the results or the figures. Therefore, the following corrections are needed, especially in Figure 3B and Figure 4:
・The cutoff values for each parameter should be stated (e.g., no cutoff value listed for High LDL cholesterol).
・List all P values as in the Table.
・Unify the description of the parameters in Table3B and Table4.

*Thank you for your valuable advice. The above points have been addressed in the figures and tables.*

2. Conclusion: Please describe what you found out from this study (e.g., cutoff value of -1.42 for both men and women, association between blood and urine equol levels only in the producers, etc.), rather than describing the strengths of the study.

*Thank you for your insightful advice. The above points have been addressed in the conclusion.*

Minor points
3. Please include the abbreviation for PSA in the abstract.

*Thank you for your insightful advice. The above points have been addressed in the abstract.*

4. Male/female and men/women are mixed in the paper. Please unify them.
*Thank you for your insightful advice. The above points have been addressed.*

5. Figure1: "Density" on the vertical axis should be changed to an appropriate term.
*Thank you for your advice. The above points have been addressed in the figure 1.*
6. Table 1: Please include the abbreviation of the parameter in the footnote as well. Or, please provide the full name of the parameter in the table.

*Thank you for your insightful advice. The above points have been addressed in table 1.*
7. Table1：Please also indicate the median age.
*Thank you for your insightful advice. The above points have been addressed in table 1.*
8. Table2: Please indicate the median age of each of the producers and non-producers. Please adjust the decimal point of the parameter to Table3.

*Thank you for your insightful advice. The above points have been addressed in table 3.*

9. Table3: Please indicate the median age of each of the producers and non-producers.

*Thank you for your insightful advice. The above points have been addressed in table 3.*

Reviewer #2: Thank you very much for allowing me to review this important research undertaking. I have some comments:

Introduction:
1. The first paragraph only accounts for a single reference. I wonder if all those information were all lifted from the same source.

*Thank you for your insightful advice. References were added in the first paragraph of the introduction.*

2. The introduction part is a bit weak. There should be a strong statement of the problem on the reasons why equol should be given priority and then transitioning to the research gap. Recent researches on epidemiological research on equol should also be stated to describe current landscape on such research topic.

*Thank you for your insightful advice. Epidemiological research on equol, statement of the problem and rationale for research objective have been added in the introduction.*

Methods
1. Were the participants asked if they were soy drinkers and the frequency of drinking soy? This is an important variable as this might have an effect on the outcome measured.
*Thank you for your insightful advice. We were not able to collect dietary assessments in this research and revised the methods and limitation sections for this.*

Discussion:
Overall, the discussion is a weak. It should discuss public health implication of the findings and how can the government address the health issue on hand. Also, strengths of the study should also be included before the limitation part of the discussion.The limitation part also lacks in details in terms of the study design limitations. Kindly improve including the objectives.

*Thank you for your valuable advice. We have addressed the points in the discussion section.*

Reviewer #3: In the study, urinary and blood isoflavones were determined in participants of annual health checkups. The associations between equol production and other various outcomes were investigated.

The findings on the relationship between blood and urinary levels are not surprising even though they may examine them in each sex and equol metabolic phenotype.

The cut-off value for equol phenotype was previously proposed by Ideno et al. and the authors in this study employed them and no additional investigation was conducted. Thus they found the threshold around -1.4 in log-scale and it does not give new insights.

The authors appeal that they found beneficial effects of equol on PSA in male population. In this study, the authors conducted comparisons of various outcomes other than PSA, and thus it is likely a statistical chance. Indeed, other outcomes showed statistically significant differences between equol phenotypes, but the authors focused only on PSA, this is cherry-picking and p-value hacking.

Background of participants is highly unclear.
During Jun 2016¬ to Dec 2017, only 466 persons visited the health screening? Total number of examined persons should be given. In addition, the participants were recruited at Kyoto University but the IRB approval was provided by University of Tokyo. Why?
Further, ranges of ages are too wide to evaluate the possible subclinical effects.

*Thank you for your advice. These participants were the members of the facility that we carried out research, not the entire hospital, therefore, the number is low. Although the study was conducted at the Kyoto University Hospital, we need to submit the proposal to a third-party ethical review board according to the regulations of Kyoto University Hospital for the use of secondary data by researchers from different affiliations. We have added those points in the method section. We have deleted the age ranges from the table as the numbers of subjects in each age range were too small if we change to narrower ranges of ages.*

Sample size estimation was not appropriate. The effect size was only based on the phenotype differences between genders and not applied for other outcomes. Especially, 37% difference between genders is not plausible.
In addition, various outcomes examined in this study are affected by other background characteristics of participants. Absence of the information is critical to investigate potential relationship between equol phenotype and those outcomes.
*Thank you for your valuable advice. We have added those points as limitations of our study.*

Other points:

Why blood isoflavone levels should be corrected by creatinine?

*Thank you for your valuable advice. We have corrected these levels.*

Significant digits should be unified through the texts and tables.

*Thank you for your valuable advice. We have unified them.*

The authors stated “However, it would not affect the results of this epidemiological study significantly”, but this does not make sense.
*Thank you for your valuable advice. We have added those points as limitations of our study.*

Figure 3A should be with error bars.
*Thank you for your valuable advice. We have added error bars in figure 3A.*

In Figure 3B and Figure 4, numbers in each category and p-values should be given.

*Thank you for your valuable advice. We have added those points in figure 3B and figure 4.*

6. PLOS authors have the option to publish the peer review history of their article ([what does this mean?](https://journals.plos.org/plosone/s/editorial-and-peer-review-process#loc-peer-review-history)). If published, this will include your full peer review and any attached files.


If you choose “no”, your identity will remain anonymous but your review may still be made public.


**Do you want your identity to be public for this peer review?** For information about this choice, including consent withdrawal, please see our [Privacy Policy](https://www.plos.org/privacy-policy).

Reviewer #1: No

Reviewer #2: No

Reviewer #3: No

[NOTE: If reviewer comments were submitted as an attachment file, they will be attached to this email and accessible via the submission site. Please log into your account, locate the manuscript record, and check for the action link "View Attachments". If this link does not appear, there are no attachment files.]

While revising your submission, please upload your figure files to the Preflight Analysis and Conversion Engine (PACE) digital diagnostic tool, <https://pacev2.apexcovantage.com/>. PACE helps ensure that figures meet PLOS requirements. To use PACE, you must first register as a user. Registration is free. Then, login and navigate to the UPLOAD tab, where you will find detailed instructions on how to use the tool. If you encounter any issues or have any questions when using PACE, please email PLOS at [figures@plos.org](mailto:figures@plos.org). Please note that Supporting Information files do not need this step.
