# Supplementary material for: Comparison of blood and urine concentrations of equol by LC‒MS/MS method and factors associated with equol production in 466 Japanese men and women
Source: PLoS One. 2024 Mar 27;19(3):e0288946. doi: 10.1371/journal.pone.0288946 (PMC10971664; doi:10.1371/journal.pone.0288946)
Supplement: Attachment — Submitted filename: Response to reviewers_PONE-D-23-20874_V3.docx [file pone.0288946.s006.docx]

Reviewer #3: Authors’ responses are not by point by point. I cannot understand what revisions were made to my comments.

*We apologize for not addressing point by point. Let us respond your previous comments as follows.*

**Responses for the previous comments by Reviewer #3.**

Reviewer #3: In the study, urinary and blood isoflavones were determined in participants of annual health checkups. The associations between equol production and other various outcomes were investigated.

The findings on the relationship between blood and urinary levels are not surprising even though they may examine them in each sex and equol metabolic phenotype.The cut-off value for equol phenotype was previously proposed by Ideno et al. and the authors in this study employed them and no additional investigation was conducted. Thus, they found the threshold around -1.4 in log-scale and it does not give new insights.

*Thank you for your insightful comments. We have mentioned the distinction in the discussion as follows.*

*" Previously, this cut-off value was reported in women participants only [34]. Therefore, this is the first study that could reproduce the same results in both men and women. Additionally, we found that urinary and serum equol concentrations were significantly correlated in equol producers but not in nonproducers. This also highlighted the important concept that it could be difficult to differentiate the equol producer phenotypes relying on either absolute serum or urinary equol concentrations."*

The authors appeal that they found beneficial effects of equol on PSA in male population. In this study, the authors conducted comparisons of various outcomes other than PSA, and thus it is likely a statistical chance. Indeed, other outcomes showed statistically significant differences between equol phenotypes, but the authors focused only on PSA, this is cherry-picking and p-value hacking.

*Thank you for your comment. We have discussed other outcomes as follows.*

*"* *Studies on the benefits of soy isoflavones have yielded inconsistent results. This could be most likely due to the variations in equol producer phenotypes. Even in the studies that assessed the equol producer phenotypes, some results failed to reach statistical significance due to small sample sizes. For example, in this study, women equol nonproducers tended to have higher LDL cholesterol, triglyceride, high sensitivity C-reactive protein and uric acid levels, but the results were not statistically significant. However, in our previous study on 743 healthy women, equol producers in their 50s and 60 s, the age groups with declining estrogen levels, had favorable blood levels of lipids, uric acid, bone resorption markers, high sensitivity C-reactive protein, and homocysteine [40]. These positive effects were due to the estrogenic and antioxidant action of equol."*

Background of participants is highly unclear.
During Jun 2016¬ to Dec 2017, only 466 persons visited the health screening? Total number of examined persons should be given. In addition, the participants were recruited at Kyoto University but the IRB approval was provided by University of Tokyo. Why?

*Thank you for your advice. These participants were the members of the facility that we carried out research, not the entire hospital, therefore, the number is low. Although the study was conducted at the Kyoto University Hospital, we need to submit the proposal to a third-party ethical review board according to the regulations of Kyoto University Hospital for the use of secondary data by researchers from different affiliations. We have added those points in the method section as follows.*

*"Although the study was conducted at the Kyoto University Hospital, we need to submit the proposal to a third-party ethical review board according to the regulations of Kyoto University Hospital for the use of secondary data by researchers from different affiliations. Therefore, the study was approved by the Institutional Review Board of The University of Tokyo (Supplementary data file 2)."*

Further, ranges of ages are too wide to evaluate the possible subclinical effects.

*Thank you for your comment. We have deleted those age ranges in the tables 1-3.*

Sample size estimation was not appropriate. The effect size was only based on the phenotype differences between genders and not applied for other outcomes. Especially, 37% difference between genders is not plausible.
In addition, various outcomes examined in this study are affected by other background characteristics of participants. Absence of the information is critical to investigate potential relationship between equol phenotype and those outcomes.
*Thank you for your valuable advice. We have added those points as limitations of our study as follows.*

*"* *Furthermore, the effect size we used in the sample size calculation was only based on the phenotype differences between genders and not applied for other outcomes. Especially, 37% difference between genders is not plausible."*

Other points:

Why blood isoflavone levels should be corrected by creatinine?

*Thank you for your valuable advice. We have corrected these levels in the tables 1-3.*

Significant digits should be unified through the texts and tables.

*Thank you for your valuable advice. We have unified them.*

The authors stated “However, it would not affect the results of this epidemiological study significantly”, but this does not make sense.
*Thank you for your valuable advice. We have added those points as limitations of our study as follows.*

*"First, we did not have the detailed characteristics of the study participants, including medical history, anthropometric measures, and dietary habits. Therefore, that might affect the outcomes of the study. Especially the dietary habits might affect the proportion of equol producers since the isoflavone concentration in the body fluctuates with soy intake and is influenced by many other dietary factors."*

Figure 3A should be with error bars.
*Thank you for your valuable advice. We have added error bars in figure 3A.*

In Figure 3B and Figure 4, numbers in each category and p-values should be given.

*Thank you for your valuable advice. We have added those points in figure 3B and figure 4.*

Reviewer #4: Authors have appropriately addressed the comments and suggestions from the three reviewers in the first round of review. I have no more comment.

*Thank you for your comment. We really appreciate your valuable insights and suggestions.*

Reviewer #5: This study provides important information about isoflavone intake and health effects via the epidemiologic cross-sectional designed investigation. To report its achievements through Plos One, please consider and revise about comments below.

The conclusion should be included in one sentence in the abstract. You need a sentence like this. “This study suggests (or found) that ~ .”

*Thank you for your comment. We added that sentence in the abstract as follows.*

*"This study suggests a relationship between equol-producing ability and reduced risk of prostate disease as well as positive effects of equol on blood lipids and uric acid levels."*

Page 6. Please describe the abbreviation “LC/MS/MS” here (not page 9), because it was firstly used in this manuscript.

*Thank you for your comment. We described the abbreviation in page 6 as you suggested.*

Several expressions on the page 12 are not good on the Results section. Expressions such as “consistent with our definition”, “relevant in our population” must be moved to the Discussion section.

*Thank you for your comment. We moved that section to the Discussion section as you suggested.*

Page 15. 1st sentence. observation → relationship

*Thank you for your comment. We corrected the term as you suggested.*

Figure 3A. Numbers of each age range are too small. I recommend unifying 20s and 30s as well as 70s and 80s.

*Thank you for your comment. We have combined the age ranges of 30s and 40s and 70s and 80s and revised figure 3A.*

Page 23. The sentences “In addition, ~ examined in this study.” are a duplication with the first limitation.

*Thank you for your comment. We have deleted that sentence from limitation.*

Authors emphasize LC/MS/MS method such an extent to include the title and key words. Then please explain the meaning of this method in your study. It is not a newly developed or validated method in this study, and readers may be curious about this method whether any special strength or not than other methods (i.e. GC-MS or anything else).

*Thank you for your comment. We added those facts in the method section as follows.*

*"In brief, 100 µL of serum or 10-fold diluted urine was mixed with internal standards, followed by the addition of 150 µL of an β-glucuronidase enzyme solution for deconjugation (Roche Biochemical, Mannheim, Germany). Following a one-hour deconjugation reaction at 37°C, free equol, daidzein, and genistein were purified using solid-phase extraction (Oasis PRiME HLB, Waters, Milford, MA). Subsequently, liquid chromatography (LC) -tandem mass spectrometry (LCMS-8050, Shimadzu, Japan) was employed with a reverse-phase LC column (ACQUITY UPLC HSS T3, 1.8 µm, 2.1 mm × 100 mm, Waters, Milford, MA) for analysis. Data processing was conducted using Mass Hunter software (Agilent, Santa Clara, CA). The peak areas were normalized using internal standards, and the concentration of each analyte was determined through a standard curve."*

*"The advantage of LC-MS/MS over other methods such as glass chromatography-based methods for detection of isoflavones is that all the conjugated and unconjugated isoflavones and their metabolites can be separated and analysed faster and more efficiently. [35, 36]. Therefore, it has been used extensively in quantitative measurements of isoflavones in several studies　[37-41]."*

Page 24. 3rd line. Association → relationship

*Thank you for your comment. We corrected the term as you suggested.*

Reviewer #6: The article describes the investigation of the equol level in blood and urine samples from 466 Japanese men and women. Unfortunately, no information about the dietary habits are available which limits the suitability of this study. In my opinion it is important to state this limitation already in the abstract.

*Thank you for your comment. We added that sentence in the abstract as follows.*

*"* *However, lack of dietary information and disperse age groups were major drawbacks in generalizing the results of this study."*

Cut-off value: I am sorry, but I do not understand how the authors “analysed” the cut-off value. As far as I understood it, they just took it from a previous publication. Please clarify it.
*Thank you for your comment. We added the following points for what is unknown from the previous study and our hypothesis in the introduction before the objective of the study as follows.*

*"* *However, their study was conducted only among women and there was no such study among men nor reproducibility of that definition has never been tested in both sexes. We hypothesized there were differences between men and women with regards to equol producing ability, i.e., they might have different cut-off values, as well as its relationship with other biomarkers inside the body."*

Furthermore, I have a problem with the statements of the authors drawing conclusions with the health data. First of all, there are no information about the dietary habits of the participants, so whether or not equol was detected in blood or urine greatly depends on the last consumption of isoflavone containing food. Secondly, the number of participants in the different age groups were rather limited. In my opinion, it is important to point out these limitations already in the abstract.

*Thank you for your comment. We have added that sentence in the abstract and conclusion section as follows.*

*"* *However, lack of dietary information and disperse age groups were major drawbacks in generalizing the results of this study."*

*"However, we need more robust clinical trials in the representative samples of different age groups including dietary assessments to determine the health benefits of equol in both men and women."*

The authors provided the minimal data set, but without an explanation, it is not clear to me what the individual columns are standing for.

*Thank you for your comment. We have added the explanation of the columns in the minimal data set in a new sheet.*

#Please check the English language in the whole manuscript, e.g., sometimes you use articles where there should be no article (page 12, line 8 – there should not be an article before “between” “men and women”).

*Thank you for your comment. We have corrected that point as you suggested.*

Keywords: As far as I have learnt it the key words should be different than the words used in the title.

*Thank you for your comment. We have changed the key words as you suggested.*

Page 6 at the end of the page: What do you mean with “LC/MS/CS”? Do you mean “LC-MS/MS”?

*Thank you for your comment. We have corrected the abbreviation as you suggested.*

Page 7 – line 2: I have learnt that “but” should not be used at the beginning of the sentence. Please consider using “However” instead.

*Thank you for your comment. We have corrected the vocabulary as you suggested.*

Page 9: I am missing some details on the used method. Ideally you should provide them in the manuscript itself, but at least you should provide a reference.

*Thank you for your comment. We added those facts and references in the method section as follows.*

*"In brief, 100 µL of serum or 10-fold diluted urine was mixed with internal standards, followed by the addition of 150 µL of an β-glucuronidase enzyme solution for deconjugation (Roche Biochemical, Mannheim, Germany). Following a one-hour deconjugation reaction at 37°C, free equol, daidzein, and genistein were purified using solid-phase extraction (Oasis PRiME HLB, Waters, Milford, MA). Subsequently, liquid chromatography (LC) -tandem mass spectrometry (LCMS-8050, Shimadzu, Japan) was employed with a reverse-phase LC column (ACQUITY UPLC HSS T3, 1.8 µm, 2.1 mm × 100 mm, Waters, Milford, MA) for analysis. Data processing was conducted using Mass Hunter software (Agilent, Santa Clara, CA). The peak areas were normalized using internal standards, and the concentration of each analyte was determined through a standard curve."*

*"The advantage of LC-MS/MS over other methods such as glass chromatography-based methods for detection of isoflavones is that all the conjugated and unconjugated isoflavones and their metabolites can be separated and analysed faster and more efficiently. [35, 36]. Therefore, it has been used extensively in quantitative measurements of isoflavones in several studies　[37-41]."*

Page 9 – metabolic parameters: Please check, but there should always be a space before the brackets (e.g., “glucose level (FBG)”). Furthermore, in English the compound names should be written with small starting letters (e.g., estradiol). Please check this in the whole manuscript.

*Thank you for your comment. We have corrected those vocabularies as you suggested.*

Page 12 – evaluation of equol producer status: You state “we applied the same finite fixed model as the previous study”, but you do not provide a reference. Please add it here.

*Thank you for your comment. We have added the reference as you suggested.*

Page 12 – line 6: Please check, but you sometimes used “n” and sometimes “N”, please be consistent.

*Thank you for your comment. We have made "n" consistent as you suggested.*

Table 1-3: Why did you change the order in these tables? I would use always the same order since these tables state the same information, but in different groups (men & women, men and women).

*Thank you for your comment. We have unified the order of the tables as you suggested.*

In Table 1: Why did you report serum concentrations as µg/g Cr? This unit belongs to the urinary concentrations, but not to serum. In the other two tables (2 and 3) you used the unit “ng/dL”. Furthermore, please never report analytical results of “zero” as in case of estradiol. Always state “lower than the limit of detection < LOD).

*Thank you for your comment. We have corrected the units and used LOD in place of zero as you suggested.*

Please explain all used abbreviations – also “BMI” (page 23).

*Thank you for your comment. We have explained the abbreviations as you suggested.*

Supporting information file 3: Please check the HDL-cholesterol level – is there really a 10 fold difference between men and women? All other values are exactly the same between men and women

*Thank you for your comment. We have corrected the value as you pointed out.*

References: Please check the references carefully.

“in vitro”, “in vivo” should be written in general written in italics.

*Thank you for your comment. We have written them in italics as you suggested.*

Reference 5: It should be a “beta (β)” symbol not a “ß” which is a kind of “s” in German.

*Thank you for your comment. We have corrected the symbol as you suggested.*

Several times not only the year, but also the month of publication is provided. I think that it is not necessary to state the month as well.

*Thank you for your comment. We have omitted the months as you suggested.*

Reference 11: Please check – I am not sure what the number eight (8) means prior to the author names.

*Thank you for your comment. We have corrected that point as you suggested.*

Reference 43: I think that here something is missing. Please check.

*Thank you for your comment. We have added some more information as you suggested.*

Figures and Tables should be understandable without reading the manuscript itself. Therefore, please explain the used abbreviations (e.g., “EQP” and “EQNP” in Figure 2).

Figure 1: You changed the nomenclature in the whole manuscript to “men” and “women”; but in this figure you still you “male” and “female”. Please correct it here as well.

*Thank you for your comment. We have corrected that point as you suggested.*

Figure 2: Please state the units reported in theses graphs. It is not clear to me what you did. Furthermore, in each subgraph for male and female two Rsquare values are provided, but only in case of male it is specified to which group it belongs. Moreover, you changed the nomenclature in the whole manuscript to “men” and “women”; but in this figure you still you “male” and “female”. Please correct it here as well.

*Thank you for your comment. We have corrected the points as you suggested.*

Figure 3A Please check the numbers provided below the figure. In case of “20s” should it not be (0, 2) instead of (2, 0). As far as I understood it the first number belongs to equol non-producers and the second number to equal producers. Furthermore, in case of “80s”, how can you provide a standard deviation when only one person belonged to the group. Please also specify which type of standard deviation is provided.

*Thank you for your comment. We have corrected the points as you suggested and combined 20s and 30s, as well as 70s and 80s as the numbers are quite low.*

Please unify, because in Figure 3B and 4 first equol-non-producers are provided, whereas in Figure 3A you changed the order and provide first equol producers and then equol-non-producers. In my opinion it would be less confusing if the order is always the same.

*Thank you for your comment. We have corrected the points as you suggested.*
